# Identifying significant gene‐environment interactions using a combination of screening testing and hierarchical false discovery rate control

**DOI:** 10.1002/gepi.21997

**Published:** 2016-08-31

**Authors:** H. Robert Frost, Li Shen, Andrew J. Saykin, Scott M. Williams, Jason H. Moore, the Alzheimer's Disease Neuroimaging Initiative

**Affiliations:** ^1^ Departments of Biomedical Data Science and GeneticsInstitute for Quantitative Biomedical SciencesGeisel School of Medicine, Dartmouth CollegeHanoverNH 03755USA; ^2^ Center for Neuroimaging and Indiana Alzheimer's Disease CenterDepartment of Radiology and Imaging SciencesIndiana University School of MedicineIndianapolisIN 46202USA; ^3^ Division of InformaticsDepartment of Biostatistics and EpidemiologyInstitute for Biomedical InformaticsPerelman School of MedicineUniversity of PennsylvaniaPhiladelphiaPA 19104‐6021USA

**Keywords:** gene‐environment interactions, screening testing, hierarchical FDR, penalized regression

## Abstract

Although gene‐environment (G× E) interactions play an important role in many biological systems, detecting these interactions within genome‐wide data can be challenging due to the loss in statistical power incurred by multiple hypothesis correction. To address the challenge of poor power and the limitations of existing multistage methods, we recently developed a screening‐testing approach for G× E interaction detection that combines elastic net penalized regression with joint estimation to support a single omnibus test for the presence of G× E interactions. In our original work on this technique, however, we did not assess type I error control or power and evaluated the method using just a single, small bladder cancer data set. In this paper, we extend the original method in two important directions and provide a more rigorous performance evaluation. First, we introduce a hierarchical false discovery rate approach to formally assess the significance of individual G× E interactions. Second, to support the analysis of truly genome‐wide data sets, we incorporate a score statistic‐based prescreening step to reduce the number of single nucleotide polymorphisms prior to fitting the first stage penalized regression model. To assess the statistical properties of our method, we compare the type I error rate and statistical power of our approach with competing techniques using both simple simulation designs as well as designs based on real disease architectures. Finally, we demonstrate the ability of our approach to identify biologically plausible SNP‐education interactions relative to Alzheimer's disease status using genome‐wide association study data from the Alzheimer's Disease Neuroimaging Initiative (ADNI).

## 1 INTRODUCTION

Statistical gene‐environment (G× E) interactions are known to be biologically important for a wide range of human phenotypes and environmental exposures (Buil et al., 2015; Hunter, 2005; Murcray, Lewinger, & Gauderman, 2009). Knowledge of significant G× E interactions has the potential to improve our understanding of disease mechanisms, enable the creation of better risk prediction models, and guide the development of more effective prevention and treatment strategies (Aschard et al., 2012b). Motivated by these benefits, researchers have used the data generated by genome‐wide association studies (GWASs) to search for G× E interactions relative to many common human diseases. Unfortunately, few significant and replicated G× E interactions have been identified using GWAS data to date (Aschard et al., 2012a; Campa et al., 2011; Hutter et al., 2013). Although G× E interaction detection is challenged by confounding, exposure measurement error, population stratification, and G× E interaction dynamics, insufficient statistical power may be the most important factor behind the limited success researchers have had finding G× E interactions (Aschard et al., 2012b). Because GWAS data sets measure on the order of one million genetic markers, multiple hypothesis correction (MHC) has a significant impact on the power of methods that assess marginal and interaction effects using a separate statistical test per marker. Even when the sample size is large enough to identify expected marginal associations after MHC, a common requirement for GWAS data sets, G× E interactions may be missed because the power to detect interactions is much lower than the power to detect main effects for a specific sample size (Murcray, Lewinger, Conti, Thomas, & Gauderman, 2011; Mukherjee, Ahn, Gruber, & Chatterjee, 2012).

In an attempt to improve statistical power, researchers recently developed a number of new G× E interaction detection methods based on a multistage or screening‐testing framework (Dai, Kooperberg, Leblanc, & Prentice, 2012; Hsu et al., 2012; Kooperberg & Leblanc, 2008; Murcray, Lewinger, & Gauderman, 2009; Murcray, Lewinger, Conti, Thomas, & Gauderman, 2011; Wu, Chen, Hastie, Sobel, & Lange, 2009). In these methods, the set of all candidate markers is first screened according to a filter statistic computed separately for each marker, e.g., the marginal association between a marker and the phenotype. The subset of markers passing the screen at the desired threshold are then tested using the standard approach of one model per marker. The key benefit of these methods is a dramatic reduction in the number of hypotheses that must be considered for MHC. If the filter statistic used for screening is statistically independent of the test statistic used to assess G× E interactions under the null hypothesis, then type I error control is maintained with MHC performed for just the family of hypotheses associated with the markers that pass screening. Although these screening‐testing methods are statistically valid and have been shown to be more powerful than approaches that test one model per marker, they have several important drawbacks including varied performance based on the choice of filter statistic, the fact that markers are filtered and tested in separate models, and the fact that power is still impacted by MHC (the number of tested hypotheses is reduced by screening but multiple tests are performed) (Frost, Andrew, Karagas, & Moore, 2015).

To address the deficiencies of standard screening‐testing methods, we recently developed a new screening‐testing approach for G× E interaction detection, detailed in Frost et al. (2015), that uses a single elastic net penalized regression model in the screening stage following by a single unpenalized regression model in the test stage to support an omnibus test for the presence of G× E interactions. Although our new method, here dubbed sequential penalized and unpenalized regression or SPUR, was shown to be an improvement on prior screening‐testing approaches, it had two serious methodological limitations. The first restricted the number of markers that could be analyzed and the second prevented a formal test of the significance of individual interactions. Evaluation of SPUR in Frost et al. (2015) was also based on only a single, small bladder cancer data set with measurements for approximately 1,500 single nucleotide polymorphisms (SNPs) after quality control (QC).

To correct these limitations, we have extended the original SPUR method to include a score statistic‐based prescreening step and to assess the significance of individual interactions using hierarchical false discovery rate (FDR) (Yekutieli, 2008). The SPUR method has also been generalized to support both dichotomous and continuous environmental exposures. To augment the cursory evaluation in Frost et al. (2015), we have performed a more extensive and robust evaluation of both the statistical properties and practical effectiveness of the SPUR method. Specifically, the statistical properties of the method, i.e., type I error control and power, have been evaluated using a variety of simulation studies and the practical effectiveness of SPUR has been evaluated through analysis of GWAS data from the Alzheimer's Disease Neuroimaging Initiative (ADNI) (Weiner et al., 2013).

The remainder of this manuscript is organized as follows: Section 2 provides further background on data assumptions, the G× E interaction model, standard detection methods, screening‐testing approaches, and the original SPUR method. Section 3 outlines the methodological extensions made to SPUR and the design of both the simulation studies and real GWAS data analysis. Section 4 contains the simulation and real data results with a discussion in Section 5.

## 2 BACKGROUND

### 2.1 Data assumptions

It is assumed that detection of G× E interactions is based on the values of an environmental exposure, a clinical outcome or phenotype, genetic markers, and other relevant covariates for a set of subjects captured as part of a GWAS. Specifically, the GWAS data are assumed to contain measurements of the following variables for *n* subjects:

- *p* genetic markers, $G_{1},...,G_{p}$. It is assumed these are SNPs with genotype values specified using additive coding, i.e., 0, 1, or 2 corresponding to the number of minor allele copies.
- A single binary clinical endpoint, D, e.g., disease case/control status.
- A single binary or continuous environmental exposure, E.
- *c* other covariates, $C_{1},...,C_{c}$ that can be either discrete or continuous variables.

### 2.2 G× E interaction model and standard detection methods

Assuming the outcome D is binary, a G× E interaction can be defined as a departure from additivity on either a log‐odds scale or an absolute risk scale. In this paper, we assume a log‐odds scale for interactions, which can be tested using the following logistic regression model:

(1) $$\mathrm{logit}\left(P\left(D=1|G_{i},E\right)\right)=\beta_{0}+\beta_{G_{i}}G_{i}+\beta_{E}E+\beta_{G_{i}E}G_{i}E.$$

Here, the null hypothesis of no G× E interaction is $H_{0}:\beta_{G_{i}E}=0$ and the alternative hypothesis is $H_{A}:\beta_{G_{i}E}\neq 0$. The standard approach to G× E interaction detection for a binary outcome and log‐odds scale interactions fits a separate version of model (1) for each of the *p* markers. Under this approach, the statistical significance of an interaction is tested using either a likelihood ratio (LR) test comparing the model with the interaction term to the model without the interaction term or a Wald test based on the estimated ${\hat{\beta }}_{G_{i}E}$. To control either the family‐wise error rate (FWER) or FDR, the interaction *P*‐values generated via LR or Wald tests must to be adjusted using the desired MHC approach for the family of *p* separate hypothesis tests. Variations of this approach exist that also perform a separate hypothesis test for each genetic marker. These include the case‐only gene‐environment association test, the test of marginal association (i.e., testing the $\beta_{G_{i}}$ coefficient in a model without the interaction term) and the combined marginal and interaction test (i.e., a joint test of the $\beta_{G_{i}}$ and $\beta_{G_{i}E}$ coefficients)(Ziegler & König, 2010). Because all of these approaches directly test each marker using separate models, they are often referred to as “one step” or “single stage” methods in the literature. Although one step methods are easy to understand and simple to implement, they can be severely underpowered when the number of markers, *p*, is large. As mentioned above, this is due to the penalty incurred by MHC for the entire family of *p* hypotheses.

### 2.3 Screening testing

Screening‐testing methods divide the G× E interaction detection process into screening and testing stages with the goal of improving power by reducing the number of G× E interaction tests that must be considered during MHC (Dai et al., 2012; Hsu et al., 2012; Kooperberg & Leblanc, 2008; Murcray et al., 2009, 2011). In the screening stage, filter statistics are computed for all *p* markers using a separate regression model per marker. In the test stage, the significance of a G× E interaction is checked for each marker that passes the screen using the same regression models employed for one step methods, e.g., model (1). As long as the filter statistic is independent of the test statistic under the null hypothesis, type I error control is maintained for individual hypothesis tests during the test stage (Bourgon, Gentleman, & Huber, 2010). If the filter and test statistics are strongly associated under the alternative hypothesis, this approach can provide a large improvement in statistical power. As shown by Bourgon et al. (2010), control of the type I error rate for individual tests also ensures control of both the FWER and the FDR with the family of hypotheses used for the adjustment including just the small number of interactions actually tested. By similar reasoning, hierarchical FDR methods will also provide the expected FDR control when applied to just the family of hypotheses that pass the filter (see Section 3.1.2 for a more detailed discussion of hierarchical FDR methods).

Screening‐testing methods are primarily distinguished from one another by the choice of filter statistic with the marginal association filter and gene‐environment correlation filter the most common choices. For the marginal association filter, a binary outcome and log‐odds scale interactions, the filter statistic is based on the significance of the $\beta_{G_{i}}$ coefficient in the following logistic regression model:

(2) $$\mathrm{logit}\left(P\left(D=1|G_{i}\right)\right)=\beta_{0}+\beta_{G_{i}}G_{i}.$$

For the gene‐environment correlation filter and a binary exposure, the statistic is based on the significance of the $\beta_{G_{i}}$ coefficient in the following logistic regression model:

(3) $$\mathrm{logit}\left(P\left(E_{i}=1|G_{j}\right)\right)=\beta_{0}+\beta_{G_{j}}G_{j}.$$

If the environmental exposure is continuous, the following model is used instead:

(4) $$P\left(E_{i}=1|G_{j}\right)=\beta_{0}+\beta_{G_{j}}G_{j}.$$

### 2.4 SPUR method

**Figure 1:**
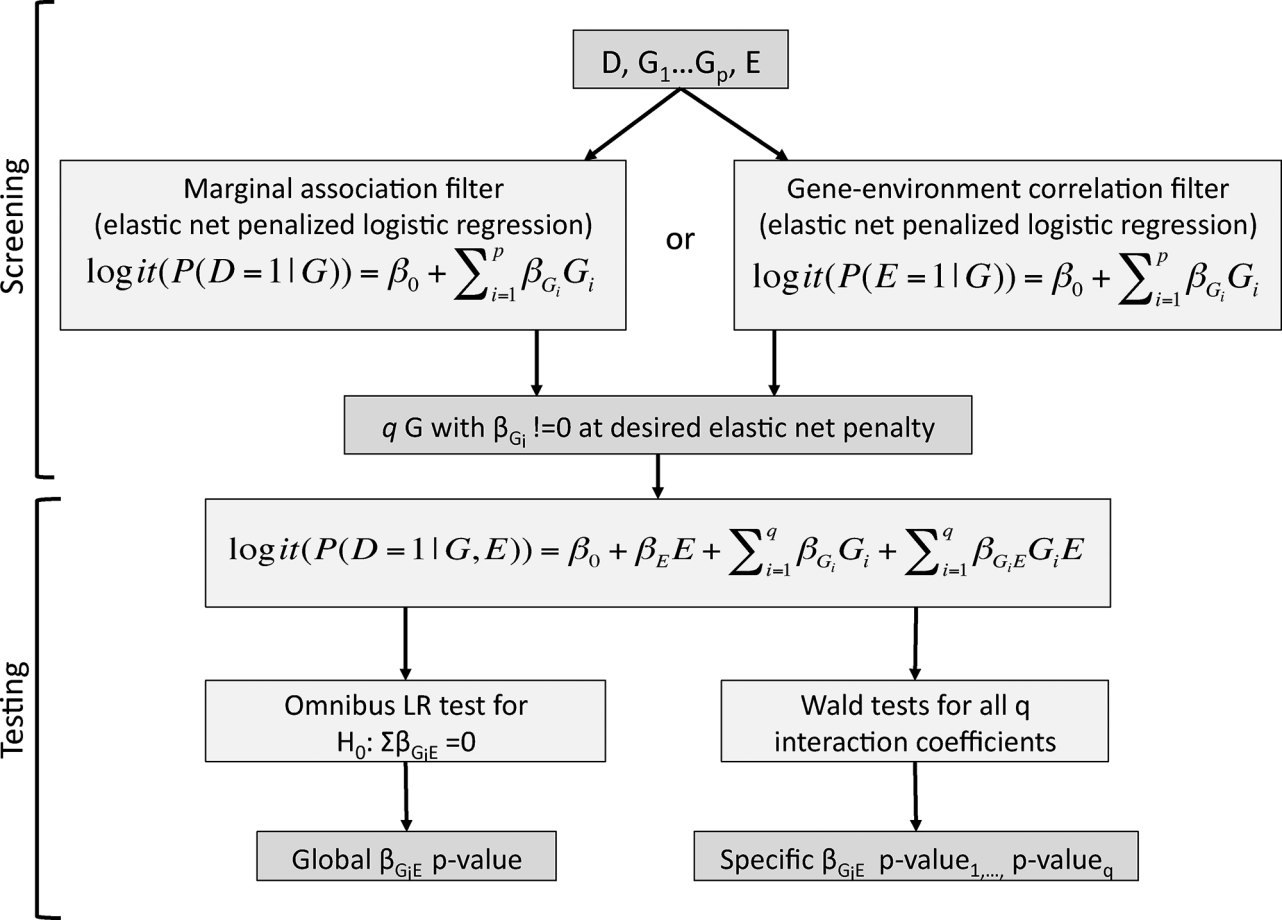
Workflow for the SPUR screening‐testing G× E detection method as presented in Frost et al. (2015)

The workflow for the original SPUR method, as described in Frost et al. (2015), is illustrated in Figure 1. To simplify notation, covariates are not included in this flow chart or in the regression models below. As shown in Figure 1, a single penalized multiple logistic regression model with predictors for all *p* markers is fit in the screening stage. For the marginal association filter, this model takes the following form:

(5) $$\mathrm{logit}\left(P\left(D=1|G\right)\right)=\beta_{0}+\sum_{i=1}^{p}\beta_{G_{i}}G_{i}.$$

For the gene‐environment correlation filter, a penalized multiple logistic regression model of the following form is used:

(6) $$\mathrm{logit}\left(P\left(E=1|G\right)\right)=\beta_{0}+\sum_{i=1}^{p}\beta_{G_{i}}G_{i}.$$

Models (5) and (6) are both fit using an elastic net (Zou & Hastie, 2005) penalty, which performs coefficient estimation under both L1, i.e., LASSO, and L2, i.e., ridge, penalties. This corresponds to maximization of the following objective function:

(7) $$\begin{array}{c} -\frac{\text{log}(L\left(\beta_{1},...,\beta_{p}|G\right)}{n}+\lambda \left(\frac{1-\alpha }{2}\sum_{i=1}^{p}\beta_{G_{i}}^{2}+\alpha \sum_{i=1}^{p}\left|\beta_{G_{i}}\right|\right), \end{array}$$

where α is the elastic net mixing parameter (α=1 corresponds to only LASSO penalization and α=0 corresponds to only ridge penalization). Selection of the elastic net penalty parameter λ can be done based on cross‐validation or to generate a specific number of nonzero coefficients. Note that if marginal terms for additional covariates are included in either model (5) or model (6), they are excluded from penalization. In Frost et al. (2015), α was set to 0.999 to provide estimation stability via a small L2 penalty (Friedman, Hastie, & Tibshirani, 2010) and λ was set so that the number of nonzero coefficients would give a ratio of observations to predictors in the test stage model of 7, per the recommendation of Vittinghoff and McCulloch (2007) for multiple logistic regression.

The test stage model for SPUR is an unpenalized multiple logistic regression model with marginal and interaction terms for all genetic markers that had nonzero coefficients in the penalized screening stage mode l (marginal terms for additional covariates are typically also included in this model):

(8) $$\begin{array}{ccc} logit(P(D=1|G,E)) & = & \beta_{0}+\beta_{E}E+\sum_{i=1}^{q}\beta_{G_{i}}G_{i}\\ & & +\sum_{i=1}^{q}\beta_{G_{i}E}G_{i}E. \end{array}$$

The primary statistical test performed on this model is an omnibus LR test comparing the model with interaction terms to the model without interactions terms. This tests the null hypothesis that all of the interaction coefficients are 0. Statistical tests, e.g., Wald tests, were also performed separately on the interaction coefficients for each marker in the model.

In the bladder cancer example included in Frost et al. (2015), the marginal association and gene‐environment correlation filters were evaluated in parallel, with a Bonferroni correction on the omnibus test *P*‐values for the two test stage models. Although standard G× E approaches failed to detect any statistically significant interactions in this data set, this SPUR‐based analysis successfully identified statistically significant associations between SNPs and smoking status relative to bladder cancer status that were biologically plausible based on prior research findings.

### 2.5 Limitations of original SPUR method

The version of the SPUR method described in Frost et al. (2015) had two major methodological limitations. The first limitation restricted the total number of genetic markers that could be analyzed; the second prevented a formal test of statistical significance for individual G× E interactions.

The original SPUR method required all genetic markers in the data set to be fit by a single penalized multiple logistic regression model in the screening stage. Because it is computationally impractical to fit models with more than several tens of thousands of predictors with current penalized logistic regression implementations (e.g., the R glmnet package; Friedman et al., 2010), this requirement meant that the original SPUR method could not be used with truly genome‐wide data sets measuring hundreds of thousands to millions of genetic markers.

In the original SPUR method, statistical significance was only formally assessed for the global test of the null hypothesis that none of the G× E interactions in the test stage model were significant, i.e., $H_{0}:\sum_{i=1}^{q}\beta_{G_{i}E}=0$. Although a Wald or LR test was performed for individual G× E interactions in significant test stage models, with a correction for the total number of interactions in the model, these adjusted interaction *P*‐values failed to account for the prior global test and could therefore only used as an informal guide to help researchers identify promising interactions for follow‐on investigations.

## 3 METHODS

### 3.1 SPUR extensions

**Figure 2:**
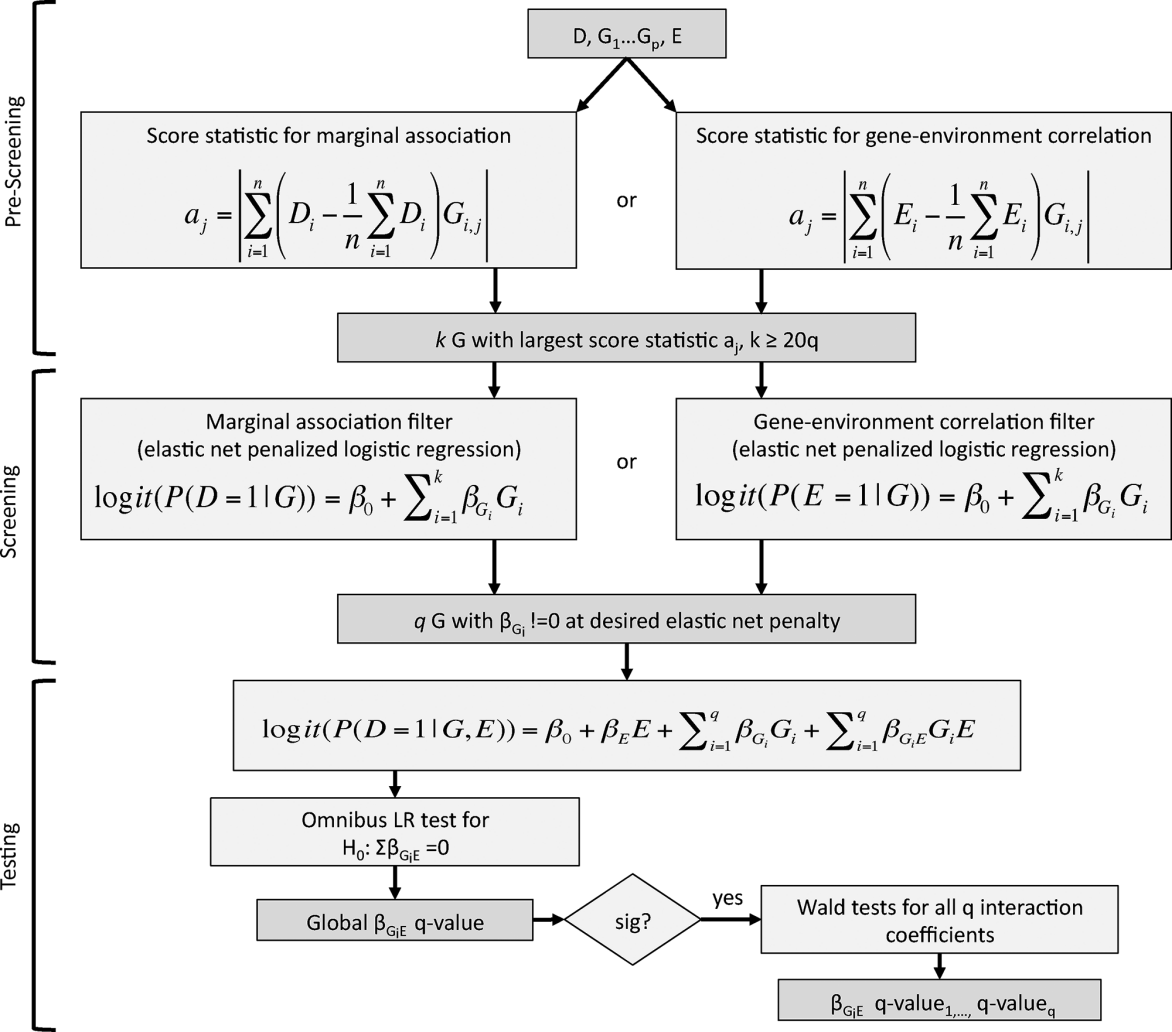
Workflow for SPUR extended to support score statistic prescreening and hierarchical FDR

To address the limitations of the original SPUR method, as outlined in Section 2.5, we have extended the technique to include both a score statistic‐based prescreening step and the hierarchical FDR assessment of individual G× E interactions. We have also generalized the formulation of the method to support dichotomous and continuous environmental exposures. Figure 2 shows the workflow for the extended SPUR method. Technical details for each extension are provided in Sections 3.1.1 and 3.1.2 and Section 3.1.3 outlines a generalized version of the SPUR regression models for continuous environmental exposures.

#### 3.1.1 Extension of SPUR to use a score statistic‐based prescreening step prior to penalized regression

To support the analysis of large GWAS data sets, i.e., data sets with hundreds of thousands to over one million markers, we have extended the SPUR method to include a prescreening step that uses a score statistic to reduce the set of measured genetic markers to a size feasible for penalized regression. This technique is motivated by the work of Wu et al. (2009) in which they advocated the use of a score statistic filter prior to fitting a LASSO‐penalized regression model for the identification of significant marginal genetic associations, gene‐gene interactions, and gene‐environment interactions. Specifically, Wu et al.'s method performed score statistic‐based filtering using the following steps (see Section 2.6 of Wu et al., 2009 for more details):

1. Compute a score statistic $a_{j}$ for each of the *p* genetic markers in the input data set. Using the notation from Section 2.1, this is defined as follows:
  (9) $$a_{j}=|\sum_{i=1}^{n}\left(D_{i}-\frac{1}{n}\sum_{i=1}^{n}D_{i}\right)G_{i,j}|.$$
2. Select the *k* genetic markers with the largest $a_{j}$. They suggest setting k=10q, where *q* represents the desired number of markers selected from the penalized regression model.
3. Fit the penalized regression model and select the LASSO penalty λ to achieve exactly *q* predictors.
4. Check that both the *q* selected markers and the k−q omitted markers satisfy the expected Karush‐Kahn‐Tucker (KKT) conditions specified by equations (5) and (6) in Wu et al. (2009).
5. If one of the omitted markers fails to satisfy the expected KKT condition, increase *k* by a factor of 2 and repeat starting at step 2.

In our extension of the SPUR method, we have adopted a simplified version of the Wu et al. approach. Specifically, we set *k* to be at least 20*q* (in practice, k≥100q is used) and skip the explicit check of the KKT conditions and potential iterative refinement of *k*. This simplification is motivated by the observation in Wu et al. (2009) that k=10q often works directly and, when it does not, one or two doublings (i.e., k∼40q) usually suffices to satisfy the KKT conditions. The score statistic‐based prescreening step included in SPUR also differs from the Wu et al. (2009) approach in the form of the score statistic, $a_{j}$. For prescreening prior to fitting penalized logistic regression model (5) for the marginal association filter, the score statistic defined in (9) is used. However, for prescreening prior to fitting penalized logistic regression model (6) for the gene‐environment correlation filter, the score statistic instead takes the form:

(10) $$a_{j}=|\sum_{i=1}^{n}\left(E_{i}-\frac{1}{n}\sum_{i=1}^{n}E_{i}\right)G_{i,j}|.$$

#### 3.1.2 Extension of SPUR to assess significance of specific G× E interactions using hierarchical FDR

To address the lack of a formal significance test for specific G× E interactions, we have extended the SPUR method to use the hierarchical FDR technique of Yekutieli (2008) to test the significance, at a specific FDR, of each G× E interactions in a test stage model with a significant omnibus test result. To support hierarchical FDR, statistical testing of model (8) is organized into a hierarchy whose top level is comprised by the hypotheses associated with the omnibus LR tests. The number of hypotheses in this level therefore corresponds to the number of distinct filter statistics evaluated; currently two for SPUR: the marginal association filter and the gene‐environment correlation filter. The children of each of these omnibus test hypotheses are the hypotheses that correspond to the Wald or LR tests on the interaction coefficients for all markers included in the test stage model. This form of hierarchy is qualitatively similar to the hierarchy used in the quantitative trait locus (QTL) example provided in Section 1.2 of Yekutieli (2008) in which the top level of the hierarchy held hypotheses corresponding to QTL tests for entire chromosomes and child levels held hypotheses corresponding to increasingly finer divisions of each chromosome. Importantly, it satisfies the requirement for interlevel independence under the null needed for hierarchical FDR, i.e., the distribution of hypotheses in a given level must still be *U*(0, 1) under *H*_0_ given rejection of the parent hypothesis.

As detailed in Yekutieli (2008), hierarchical testing of hypotheses using the Benjamini and Hochberg (1995) method at *q* within each level of the hierarchy will provide level‐specific FDR control of approximately:

(11) $$FDR=\frac{q\delta^{*}\left(d+f\right)}{d+1},$$

where $\delta^{*}$ is approximately 1, *d* is the number of discoveries made within the level (i.e., the number of hypotheses rejected at level *q*), and *f* is the number of families tested. For the bladder cancer example presented in Frost et al. (2015), f=2 (for the omnibus tests associated with the test stage models populated using the marginal association filter and gene‐environment correlation filter) and d=4 for the four SNP‐smoking interactions that had significant Wald tests at a level‐specific FDR of q=0.1. Therefore, if hierarchical FDR been employed in this case with q=0.1, approximate FDR control for the interaction‐specific Wald test results would have been achieved at a level of 0.1(2+4)/(4+1)=0.12.

#### 3.1.3 Generalization of SPUR to support continuous exposure measures

If the environmental exposure, E, is continuous rather than binary, the gene‐environment correlation filter statistic is computed using an elastic net penalized version of the following multiple linear regression model:

(12) $$P\left(E=1|G\right)=\beta_{0}+\sum_{i=1}^{p}\beta_{G_{i}}G_{i}.$$

#### 3.1.4 SPUR and FDR control

The extended SPUR method described above and illustrated in Figure 2 performs statistical tests in four distinct stages with the goal of maintaining FDR control for the tests in the last stage:

1. Score statistic prescreening.
2. Marginal association or gene‐environment correlation filtering via penalized multiple logistic regression models.
3. Omnibus test on unpenalized multiple logistic regression model.
4. Individual interaction tests.

The first two stages together filter the set of hypotheses using statistics that are independent of the logistic regression‐based G×E test statistics computed in the last two stages under *H*_0_. As detailed in Section 2.3, the fact that filtering is performed using filter statistics that are independent of the test statistics under *H*_0_ ensures that FDR control can be maintained on just the family of hypotheses that pass the filter(s). Thus, the SPUR method can provide FDR control on just a single level of postfiltering hypotheses using standard FDR methods (Benjamini & Hochberg, 1995) (e.g., the family of two omnibus tests for the test‐stage models based on the marginal association and gene‐environment correlation filters) or on a hierarchy of postfiltering hypotheses using hierarchical FDR methods (Yekutieli, 2008) (e.g., the hierarchy formed by omnibus tests at the top level followed by individual interaction tests).

### 3.2 Evaluation approach

To evaluate the extended SPUR method, we analyzed a combination of simulated and real genotype data sets. The first simulation study used a simple framework similar to that employed by Dai et al. (2012) to evaluate type I error control and power. The second simulation study also assessed type I error control and power and was based on the disease architecture models developed by Aschard et al. (2012a) for breast cancer, type 2 diabetes, and rheumatoid arthritis. Finally, GWAS data from the Alzheimer's Disease Neuroimaging Initiative (ADNI) study was used to evaluate the ability of the SPUR method to detect biologically interesting interactions between SNPs and educational attainment relative to Alzheimer's disease (AD) status.

#### 3.2.1 G× E interaction detection methods

To support comparative evaluation of the extended SPUR method, the same alternative methods used in Frost et al. (2015) were also employed to identify G× E interactions for the simulated and real data sets. For each evaluated data set, G× E interaction detection was performed using three benchmark methods:

1. One step approach (as detailed in Section 2.2 using regression model (1)).
2. Standard screening testing using marginal association filter (as detailed in Section 2.3 using regression model (2) in the screening stage and model (1) in the testing stage).
3. Standard screening testing using gene‐environment correlation filter (as detailed in Section 2.3 using either regression model (3) or (4) in the screening stage and model (1) in the testing stage).

and four variations of the extended SPUR method:

1. SPUR using just the marginal association filter. In this case, an omnibus test is not performed and the significance of G× E interactions for all markers retained after screening is assessed using Wald tests with FDR correction (according to the method of Benjamini and Hochberg (1995)).
2. SPUR using just the gene‐environment correlation filter. An omnibus test is also skipped in this case with significance of G× E interactions again assessed using FDR‐adjusted Wald tests.
3. SPUR using hierarchical FDR, as detailed in Section 3.1.2, to evaluate the marginal association filter and the gene‐environment correlation filter in parallel.
4. SPUR based on just the omnibus test for interactions considering both the marginal association filter and the gene‐environment correlation filter in parallel. Specifically, each of the G× E terms included in the test stage model is assigned the FDR *q*‐value computed for the omnibus test. The FDR adjustment is based on the family of two omnibus test *P*‐values, one for the marginal association filter and one for the gene‐environment correlation filter. To support the comparison of this approach with the other methods, the number of markers to retain in the test stage model was set equal to the number with a real G× E interaction.

For all four SPUR variations, score statistic‐based prescreening, as detailed in Section 3.1.1, was performed using k=20q, where *q* is set to be the same for all SPUR methods and for the two screening‐testing benchmark approaches. The SPUR method and all benchmark approaches were implemented in R (R Core Team, 2014).

#### 3.2.2 Simple simulation design

A simple simulation design, based on the study in Dai et al. (2012), was used to characterize the statistical properties of the extended SPUR method relative to the benchmark approaches and to highlight favorable conditions for each of the two supported filter statistics. To assess type I error control, we simulated 1,000 data sets, each containing 2,000 SNPs and one binary environmental exposure for 1,000 subjects. The SNPs were generated under an assumption of Hardy‐Weinberg equilibrium with a minor allele frequency (MAF) equal to 0.2. Similar to Dai et al. (2012), the pairwise correlation between SNPs was set to 0.1 and the environmental exposure was simulated as Bernoulli(0.5). A correlation was simulated between the first 10 SNPs and the environmental exposure with a correlation coefficient of 0.05. For each of these 1,000 data sets, the binary outcome *D* was generated according to model (8). Because the data were simulated to have no true interaction effects, all $\beta_{G_{i}E}$ coefficients were set to a value of 0. For consistency with Dai et al. (2012), the other coefficients were set as follows: $\left(\beta_{0},\beta_{E},\beta_{G_{i}}\right)=\left(-4,\text{log}\left(1.5\right),\text{log}\left(1.5\right)\right)$ for i=1,...,10, i.e., a marginal association was simulated for the first 10 SNPs. Score statistic‐based prescreening was configured to remove 1,950 of the 2,000 SNPs. The λ parameter used with the elastic net penalty for the screening stage models was set so that the number of nonzero coefficients in the estimated model, *q*, was 25. The number of markers retained after screening for the standard screening‐testing method was also set to 25 to support a comparative evaluation. The type I error rate was computed at an α=0.05 level for all benchmark method and SPUR using either the marginal association filter or the gene‐environment correlation filter, i.e., all methods that generate unadjusted *P*‐values for individual G× E interactions.

To assess statistical power, data were simulated according to two different models. Model 1 was designed to work well with the marginal association filter and model 2 was designed to favor the gene‐environment correlation filter. For each of these models, a collection of data sets was simulated using a similar approach to that described for the type I error control assessment above. For these simulated data sets, 2,000 SNPs were generated with true G× E interactions for the first five markers. Coefficient values for these interactions were set as $\beta_{G_{i}E}=log\left(3\right)$ with all other interaction coefficients set to 0. For both models the SNP‐SNP correlation was set to 0.1 and the SNP‐exposure correlation was set to 0.3. For model 1, the other coefficients were simulated as simulated as $\left(\beta_{0},\beta_{E},\beta_{G_{i}}\right)=\left(-4,\text{log}\left(1.5\right),\text{log}\left(1.5\right)\right)$ for i=1,...,5 and the SNP‐exposure correlation was 0 except for the second five SNPs, i.e., only SNPs with a true G× E interaction had a marginal association and none of the SNPs correlated with the exposure had a true interaction. For model 2, the other coefficients were simulated as simulated as $\left(\beta_{0},\beta_{E},\beta_{G_{i}}\right)=\left(-4,\text{log}\left(1.5\right),\text{log}\left(3\right)\right)$ for i=6,...,10 and the SNP‐exposure correlation was 0 except for the first five SNPs, i.e., only SNPs with a true interaction were correlated with the exposure and none of the SNPs with a marginal association had a true interaction. Score statistic‐based prescreening was configured to remove 1,900 of the 2,000 SNPs. The λ parameter used with the elastic net penalty for the screening stage models was set so that the number of nonzero coefficients in the estimated model, *q*, was 5. Standard screening‐testing methods were also configured to retain five SNPs after the first stage. Statistical power for all SPUR variations and benchmark methods was computed as the proportion of the *q* SNPs with G× E interactions that were identified as significant according to an FDR *q*‐value of 0.1.

#### 3.2.3 Disease‐based simulation design

To understand the relative performance of the SPUR method in a more realistic scenario, we also assessed type I error control and power on data sets simulated according to the genetic architectures of breast cancer, type 2 diabetes, and rheumatoid arthritis. This disease‐based simulation design was based on the approach of Aschard et al. (2012a) for evaluating the impact of gene‐gene and gene‐environment interactions on disease risk predication. Specifically, we customized Aschard's original simulation code to support disease‐based simulation scenarios for the comparative evaluation of G× E detection methods. For each of the three disease architectures, type I error control and power were assessed using separate simulation designs. In both cases, 1,000 data sets were generated for each disease with each data set containing 1,000 subjects with measurements for the set of known risk SNPs and known environmental risk factors, as detailed for each disease in the supplementary material for Aschard et al. (2012a), and a set of additional SNPs with a MAF drawn from *U*(0.05, 0.95) and no association with the outcome. No correlation was simulated between markers, between exposures, or between markers and exposures. The binary outcome variable, reflecting disease case‐control status, was simulated according to the model represented by equation (2) in Aschard et al. (2012a). To perform G× E detection in the context of a case‐control study, we simulated a large number of subjects and then sampled cases and controls from this collection to achieve 500 cases and 500 controls in each data set (this is similar to the approach used by GCTA tool (Yang, Lee, Goddard, & Visscher, 2011)). For type I error control simulation, the number of additional SNPs was set to 2,000, no G× E interactions were simulated, SPUR prescreening was set to remove 1,950 SNPs, and a total of 20 SNPs were retained in the final test stage models. For power simulation, 2,000 additional SNPs were created, G× E interactions were simulated for the five known risk SNPs with the largest relative risk for each disease with the interaction effect $\gamma_{l,m}$ in the Aschard model set to ±log(1.5) (Aschard originally simulated the interaction effect so that $Prob(\gamma_{l,m}<\text{log}\left(2\right))=0.95$), SPUR prescreening was set to remove 1,900 SNPs and a total of five SNPs were retained in the final test stage models. All five interactions were generated using a fixed environmental exposure for each disease. For breast cancer this was the number of previous biopsies, physical activity was used for type 2 diabetes, and breast feeding was used for rheumatoid arthritis.

#### 3.2.4 ADNI evaluation design

The ADNI (Weiner et al., 2013) is public‐private partnership started in 2003 with the goal of identifying biomarkers for use in AD clinical trials. During the three phases of the study conducted to date, a range of imaging, genetic, and clinical measurements have been made on study subjects with different levels of cognitive impairment (normal, early mild cognitive impairment, mild cognitive impairment, late mild cognitive impairment, and early AD). For up‐to‐date information, see www.adni‐info.org. For the evaluation of the extended SPUR method, we used clinical and genotyping data from the first phase of ADNI. For this analysis, subjects with either mild cognitive impairment or AD were considered cases and controls were cognitively normal subjects. QC and preprocessing was carried out using PLINK 1.9 (Chang et al., 2015) and involved retaining just non‐Hispanic Caucasian subjects (to eliminate population stratification issues), removing subjects missing more than 5% of the SNPs, removing all nonautosomal SNPs, removing all SNPs with a Hardy‐Weinberg test of equilibrium *P*‐value $<1\times 10^{-4}$, removing all SNPs with MAF of less than 0.01, and removing all SNPs with any missing measurements. This last QC step was quite conservative but enabled the analysis to proceed without the potential bias of an imputation method; a trade‐off we considered appropriate because the goal of this analysis was not to maximize the number of findings but rather comparative analysis of G× E detection methods. After all of these preprocessing and QC steps, 572 subjects (412 cases and 160 controls) and 398,230 SNPs, specified using additive coding, remained in the data set.

Analysis of the ADNI data focused on interactions between the number of minor allele copies for each SNP and years of educational attainment relative to case/control status. The decision to focus on SNP‐education interactions was based on prior research demonstrating a strong association between educational attainment and AD risk, progression, and severity (Amieva et al., 2014; Shpanskaya et al., 2014) and the fact that the only significant SNP‐education interactions found to date involve the APoE locus (Cook & Fletcher, 2015). Because the environmental exposure, years of educational attainment, is a continuous variable, model (12) was used to compute the gene‐environment correlation filter statistic for both standard screening testing and SPUR. Other covariates included in the regression models were age, gender, and APOE ε4 status. The three benchmark methods were implemented using PLINK logistic and linear regression functionality. The SPUR score statistic‐based prescreening step, implemented using a PLINK R (R Core Team, 2014) plug‐in, was configured to retain 20,000 of the 398,230 SNPs remaining after QC. For all two‐stage models (i.e., standard screening testing and SPUR), the number of SNPs included in the test stage model to set to 36 based on the Vittinghoff and McCulloch (2007) guideline of a 7‐to‐1 observation‐to‐predicator ratio for multiple logistic regression.

## 4 RESULTS

### 4.1 Simple simulation results

As shown in Table 1, type I error control for the simulation study detailed in Section 3.2.2 was very good for all of the benchmark methods and acceptable for SPUR using both filter statistics. As expected, the one step method had very poor statistical power for both models relative to the other benchmark and SPUR approaches. For the simulation model designed to work best with the marginal association filter, i.e., model 1, the methods that used the gene‐environment correlation filter were unable to identify any G× E interactions. Although both standard screening testing and SPUR worked well using the marginal association filter for model 1, the power of SPUR (0.697) was clearly superior to standard screening testing (0.381). For the simulation model designed to work well with the gene‐environment correlation filter, i.e., model 2, the gene‐environment correlation filter provided the best power. Although the power for the marginal association filter was lower in this case, the difference was less dramatic than with model 1. For model 2, the SPUR method had superior power over standard screening testing for both filter types (0.312 vs. 0.244 for the marginal association filter and 0.665 vs. 0.590 for the gene‐environment correlation filter).

**Table 1:** Estimated type I error rates at α=0.05 and α=0.01 and power at q=0.1 for the simulation study detailed in Section 3.2.2

	Type I Error Rate		Power (*q*=0.1)	
Method	α=0.05	α=0.01	Model 1	Model 2
One step	0.051	0.010	0.019 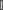	0.061 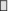
Screening testing, marginal assoc. filter	0.052	0.011	0.381 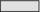	0.244 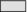
Screening testing, gene‐env. correl. filter	0.051	0.010	0.000 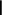	0.590 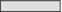
SPUR, marginal assoc. filter	0.062	0.013	0.697 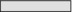	0.312 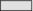
SPUR, gene‐env. correl. filter	0.057	0.012	0.000 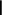	0.665 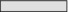
SPUR, hierarchical FDR	NA^a^	NA^a^	0.659 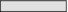	0.636 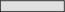
SPUR, global	NA^a^	NA^a^	0.903 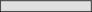	0.828 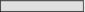

a These SPUR variants reflect the results from FDR or hierarchical FDR control so lack comparable type I error control values.

Although the type I error rate for the SPUR method is slightly inflated, the significantly improved power provides a strong motivation to use SPUR vs. existing screening‐testing approaches for hypothesis generation. Given the dramatic difference in performance between the two filters for these simulation models, it is important to note that SPUR using the hierarchical FDR approach had power superior to the best screening‐testing configuration and only slightly lower than SPUR using the optimal filter. This is a key benefit of the extended SPUR approach: a specific filter does not need to be selected and the penalty to power is minimal. Although not strictly comparable to the other methods, the power of SPUR using a global test for interactions for the test stage models for both filters had the best power of all approaches. The fact that SPUR supports a global test makes it useful in contexts where there is insufficient power to detect individual interactions, even after aggressive screening.

### 4.2 Disease‐based simulation results

As seen in Table 2, all methods provided acceptable type I error control for the three disease‐based simulation studies detailed in Section 3.2.3 with the type I error rate for SPUR very slightly inflated relative to the benchmark methods. As expected, statistical power was uniformly lower for these disease‐based simulations than for the simple simulation models. Although the decrease in power was largely due to lower marginal and interaction effect sizes, the performance of the gene‐environment correlation filter was impacted by the lack of SNP‐exposure correlation and the lack of linkage disequilibrium (LD) limited the benefit to SPUR from using a single penalized regression model during screening. Similar to the simple simulation results, the one step method had poor power relative to the two‐stage methods, SPUR was superior to standard screening testing and the global SPUR test provided the best power of all methods. It is again important to note the impressive relative power of the extended SPUR using hierarchical FDR.

**Table 2:** Estimated type I error rates at α=0.05 and power at q=0.1 for the disease‐based simulation studies detailed in Section 3.2.3

		Type I Error Rate		Power
Disease	Method	α=0.05	α=0.01	q=0.1
Breast cancer	One step	0.051	0.010	0.033 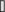
	Screening testing, marginal assoc. filter	0.0528	0.012	0.020 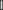
	Screening testing, gene‐env. correl. filter	0.0525	0.011	0.021 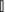
	SPUR, marginal assoc. filter	0.055	0.012	0.161 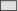
	SPUR, gene‐env. correl. filter	0.052	0.012	0.134 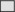
	SPUR, hierarchical FDR	NA	NA	0.187 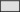
	SPUR, global	NA	NA	0.280 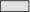
Type 2 diabetes	One step	0.052	0.011	0.019 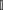
	Screening testing, marginal assoc. filter	0.052	0.010	0.100 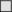
	Screening testing, gene‐env. correl. filter	0.051	0.010	0.015 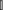
	SPUR, marginal assoc. filter	0.056	0.012	0.081 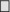
	SPUR, gene‐env. correl. filter	0.055	0.011	0.015 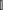
	SPUR, hierarchical FDR	NA	NA	0.074 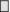
	SPUR, global	NA	NA	0.084 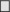
Rheumatoid arthritis	One step	0.051	0.010	0.012 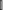
	Screening testing, marginal assoc. filter	0.51	0.010	0.157 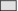
	Screening testing, gene‐env. correl. filter	0.050	0.010	0.042 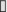
	SPUR, marginal assoc. filter	0.055	0.011	0.144 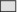
	SPUR, gene‐env. correl. filter	0.055	0.011	0.034 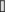
	SPUR, hierarchical FDR	NA	NA	0.126 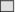
	SPUR, global	NA	NA	0.207 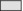

### 4.3 ADNI results

Table 3 contains the 10 most significant education‐SNP interactions computed using each of the benchmark methods for the ADNI data detailed in Section 3.2.4. As seen in the table, neither the one step method nor screening testing using the marginal association filter identified any significant interactions after MHC. Screening testing using the gene‐environment correlation filter found only a single significant interaction at the *q*=0.1 level (rs580539, located in the intron region of uncharacterized gene KIAA1211).

**Table 3:** Ten most significant education‐SNP interactions computed for the ADNI data using the one step and standard screening‐testing methods

Method	dbSNP ID	${\hat{\beta }}_{GE}$	*P*‐value	FDR
One step	rs4600636_T	4.006	$6.166\times 10^{-5}$	0.9999452
	rs513136_A	−3.816	$1.356\times 10^{-4}$	0.9999452
	rs4412_T	3.812	$1.378\times 10^{-4}$	0.9999452
	rs4709612_C	3.801	$1.444\times 10^{-4}$	0.9999452
	rs9360610_C	3.757	$1.720\times 10^{-4}$	0.9999452
	rs1554261_C	−3.753	$1.744\times 10^{-4}$	0.9999452
	rs10083119_A	3.749	$1.777\times 10^{-4}$	0.9999452
	rs1400826_G	−3.737	$1.864\times 10^{-4}$	0.9999452
	rs3763159_A	3.727	$1.937\times 10^{-4}$	0.9999452
	rs9351951_A	3.727	$1.937\times 10^{-4}$	0.9999452
Screening testing (marginal assoc. filter)	rs4678623_T	−2.3760	0.01748	0.4372200
	rs4771568_A	−2.2520	0.02429	0.4372200
	rs11856891_C	−1.7700	0.07667	0.7074000
	rs2298540_G	−1.7260	0.08438	0.7074000
	rs10892831_T	1.5490	0.12150	0.7074000
	rs6801268_T	1.5040	0.13260	0.7074000
	rs793291_A	−1.4400	0.14980	0.7074000
	rs3849196_G	1.4140	0.15720	0.7074000
	rs727735_A	−1.3290	0.18390	0.7164000
	rs10506854_G	−1.2840	0.19900	0.7164000
Screening testing (gene‐env. correl. filter)	rs580539_A	3.163	0.001563	0.0562680
	rs7532749_T	2.611	0.009021	0.1141560
	rs2785821_A	2.593	0.009513	0.1141560
	rs9316649_A	2.391	0.016800	0.1399200
	rs2976189_A	−2.268	0.023320	0.1399200
	rs2954347_G	−2.268	0.023320	0.1399200
	rs2423360_G	2.125	0.033560	0.1725943
	rs3807530_A	−2.027	0.042630	0.1836831
	rs4309408_G	1.946	0.051640	0.1836831
	rs11244744_A	1.921	0.054700	0.1836831

Table 4 contains the 20 most significant education‐SNP interactions computed using the extended SPUR method. The omnibus test *q*‐values are computed via the FDR adjustment of the two LR tests comparing the unpenalized test stage logistic regression models without interaction terms to the models with interaction terms. The SNP‐specific interaction FDR values are computed according to the hierarchical FDR procedure outlined in Section 3.1.2. Although the omnibus test results were significant for the test stage models associated with both the marginal association filter and the gene‐environment correlation filter, only the marginal association filter had individual interactions that were significant at a *q*‐value of 0.1 after hierarchical FDR adjustment. If identified in dbSNP (Sherry et al., 2001), the genes associated with the 20 most significant education‐SNP interactions for the marginal association model are listed in the table. Importantly, six of the 10 genes associated with interactions significant at q≤0.1 have a known or biologically plausible association with AD; these genes (TOMM40, TXNL1, GRIA1, SULF1, SPATA13, MIR633) are marked in bold in the table. TOMM40 is located near the APOE locus and has a well‐known association with both AD and Parkinson's disease (Gottschalk et al., 2014) (SNP rs2075650‐G also has a known association with AD; Middelberg et al., 2011). A biologically plausible link exists between TXNL1 and AD because of TXNL1's role in glucose metabolism and the cellular response to sugar starvation stress (Jiménez, Pelto‐Huikko, Gustafsson, & Miranda‐Vizuete, 2006) and the close association between AD and glucose metabolism dysfunction (Chen & Zhong, 2013; Liang et al., 2008). GRIA1 is associated with AD due to its role in synaptic plasticity (Falsafi et al., 2014). SULF1 is related to heparin sulfate, which is linked to the amyloid beta plaques characteristic of AD (Hosono‐Fukao et al., 2012). SPATA13 has a plausible association with AD because of its enriched expression within the central extended amygdala (Becker et al., 2008) and the link between atrophy of the amygdala and early AD onset and severity of symptoms (Poulin et al., 2011). MicroRNA MIR633 was found to be deregulated in the prefrontal cortex of late onset AD patients (Lau et al., 2013).

**Table 4:** Twenty most significant education‐SNP interactions computed via the extended SPUR method

Marginal					Correlation			
LR *q*‐value: 0.000232					LR *q*‐value: 0.03673			
dbSNP ID	Associated gene	${\hat{\beta }}_{GE}$	*P*‐value	FDR	dbSNP ID	${\hat{\beta }}_{GE}$	*P*‐value	FDR
rs2346567_G	LOC105373456	2.13	0.000731	0.0323	rs17586724_C	0.381	0.00705	0.239
rs7805350_T	FAM188B	2.35	0.00115	0.0323	rs1052242_T	0.315	0.0105	0.239
**rs2075650_G**	**TOMM40**	−1.33	0.00403	0.0705	rs4349644_G	−0.267	0.0147	0.239
rs573399_A	**TXNL1**	−1.26	0.00949	0.0771	rs4869558_T	−0.259	0.0227	0.266
rs11726692_G	RNF150	1.48	0.0102	0.0771	rs4779674_A	−0.336	0.0345	0.29
rs10070086_G	**GRIA1**	1.4	0.0118	0.0771	rs1229658_A	−0.33	0.039	0.29
rs1530241_A	**SULF1**	−0.791	0.0127	0.0771	rs10924293_G	−0.274	0.0602	0.377
rs2861545_G	**SPATA13**	−0.976	0.0132	0.0771	rs12822144_A	0.203	0.089	0.432
rs917924_T	Intergenic	−0.9	0.0187	0.0934	rs12679472_C	0.228	0.0907	0.432
	(LOC100288868, RPL21P46)							
rs7216013_A	Intergenic	1.62	0.0204	0.0934	rs9394169_A	−0.18	0.117	0.497
	(LOC100128712, **MIR633**)							
rs17027976_G	‐	−1.14	0.0269	0.106	rs2055407_A	0.207	0.138	0.524
rs12046563_G	PPIH	−0.851	0.0292	0.106	rs9294723_A	0.159	0.15	0.524
rs7330772_T	LOC101927437	0.839	0.0305	0.106	rs1929820_T	0.154	0.165	0.529
rs2249508_C	‐	0.643	0.0363	0.116	rs10513055_C	−0.17	0.198	0.532
rs9979680_G	‐	0.989	0.0418	0.124	rs6041429_T	0.154	0.205	0.532
rs2254595_G	FAM3C	−0.535	0.0545	0.149	rs1424976_T	−0.202	0.206	0.532
rs7574256_G	‐	0.758	0.0573	0.149	rs2119380_C	−0.14	0.234	0.562
rs10494515_A	AXDND1	0.497	0.093	0.219	rs2305252_A	−0.148	0.256	0.562
rs9595108_A	‐	0.746	0.0966	0.219	rs10177104_A	−0.137	0.261	0.562
rs4747019_A	LRRC20	0.58	0.0996	0.219	rs9681094_T	−0.123	0.321	0.63

SNPs with published associations in NHGRI‐EBI GWAS Catalog (Welter et al., 2014) are marked in bold. Genes associated with significant education‐SNP interactions, at q≤0.1, that have a plausible AD association are marked in bold.

## 5 DISCUSSION

Biologically meaningful gene‐environment interactions exist for many common human diseases and other clinically relevant phenotypes (Buil et al., 2015; Hunter, 2005; Murcray et al., 2009). If correctly identified and characterized, these interactions offer important insights into disease etiology, the design of predictive models, and effective treatment and prevention approaches. Unfortunately, poor statistical power for interaction detection has limited G× E interaction findings based on GWAS data (Aschard et al., 2012b).

In an attempt to improve G× E interaction detection power, researchers developed a number of two‐stage or screening‐testing approaches (Dai et al., 2012; Hsu et al., 2012; Kooperberg & Leblanc, 2008; Murcray et al., 2009, 2011; Wu et al., 2009). These methods divide the analysis into a screening stage, in which the set of potential markers is filtered, and a test stage, in which the markers that pass screening are evaluated for G× E interactions. Although a major improvement over approaches that test each potential interaction using a separate model, the first generation of screening‐testing methods have an important drawback, namely that separate models are used for each marker during both screening and testing. This is problematic in the presence of marker LD and also necessitates some level of MHC during the testing stage.

To address the shortcomings of early screening‐testing methods, we recently created a new screening‐testing approach for G× E interaction detection ( SPUR, reviewed in Section 2.4) that uses a single penalized model in the screening stage and a single unpenalized model in the testing stage to enable an omnibus test for G× E interactions and the parallel evaluation of multiple filter statistics. Despite promising evaluation results in Frost et al. (2015), the original SPUR method has two serious limitations: it cannot be applied to truly genome‐wide data sets and it cannot formally assess the significance of individual G× E interactions. Furthermore, statistical properties of the method (i.e., type I error rate and power) were not characterized in Frost et al. (2015) and real data evaluation was based on just a small bladder cancer data set.

In this paper, we have described an extended and generalized version of our SPUR method that corrects the methodological limitations of the original technique. To support the analysis of data sets with hundreds of thousands to millions of genetic markers, we added a score statistic‐based prescreening step, detailed in Section 3.1.1, that can reduce the initial set of markers to a size practical for penalized multiple logistic regression. As demonstrated by the simulation results in Sections 4.1 and 4.2, this prescreening technique does not adversely impact type I error control or the superior statistical power of SPUR relative to benchmark methods. For analysis of the ADNI GWAS data set, this prescreening method was used to reduce the number of SNPs from approximately 400,000 to just 20,000, illustrating the practical utility and computational efficiency of the approach. To support the formal statistical assessment of individual G× E interactions in the test stage models, we incorporated a hierarchical FDR approach, detailed in Section 3.1.2. This approach has the important benefit of enabling the parallel assessment of multiple filter statistics. In this scenario, the omnibus test results for the test stage logistic regression models associated with each filter form the top level in the hierarchy and the Wald or LR tests for individual G× E interaction terms in the test stage model form the bottom level. As shown by the simulation results, the hierarchical FDR method incurs only a small loss in power relative to SPUR using just the most optimal filter statistic. Finally, to support analysis of a broader class of experimental data, we generalized SPUR to handle both continuous and dichotomous environmental exposures. This generalized support was specifically leveraged to enable analysis of the ADNI GWAS data for interactions between years of education and SNPs relative to case/control status.

This paper also contains a much more thorough evaluation of the SPUR method using simulation studies to assess type I error control and power and a large GWAS data set to assess practical utility. Both the simple simulation design and the simulations based on the genetic architectures of breast cancer, type 2 diabetes, and rheumatoid arthritis demonstrate that the extended SPUR method has acceptable type I error control and superior power to detect known G× E interactions relative to competing approaches. The two models used with the simple simulation design also highlighted the dramatic impact that the filter statistic can have on overall performance of screening‐testing methods. This fact makes the hierarchical FDR support in the extended SPUR method especially useful because it allows both filters to be evaluated in parallel with only a small loss in power. Analysis of the ADNI GWAS data shows that the extended SPUR method can be used to analyze truly genome‐scale data sets. Importantly, the extended SPUR method was able to identify statistically significant, biologically plausible, and previously unreported education‐SNP interactions despite the relatively small sample size of the ADNI data.

In future work, we plan to use the SPUR method to search for G× E interactions in a wider range of GWAS data sets and explore methodological enhancements such as support for higher order interactions and the concurrent evaluation of different marker codings and interaction scales.
